# Motion tracking and gait feature estimation for recognising Parkinson’s disease using MS Kinect

**DOI:** 10.1186/s12938-015-0092-7

**Published:** 2015-10-24

**Authors:** Ondřej Ťupa, Aleš Procházka, Oldřich Vyšata, Martin Schätz, Jan Mareš, Martin Vališ, Vladimír Mařík

**Affiliations:** Department of Computing and Control Engineering, University of Chemistry and Technology in Prague, Technická 5, 166 28 Prague 6, Czech Republic; Department of Neurology, Charles University, Sokolská 581, 500 05 Hradec Kralove, Czech Republic; Czech Institute of Informatics, Robotics and Cybernetics, Czech Technical University, Zikova 1903/4, 166 36 Prague 6, Czech Republic

**Keywords:** Image and depth sensors, Gait disorders, Motion features, Video processing, MS Kinect, Classification, Parkinson’s disease

## Abstract

**Background:**

Analysis of gait features provides important information during the treatment of neurological disorders, including Parkinson’s disease. It is also used to observe the effects of medication and rehabilitation. The methodology presented in this paper enables the detection of selected gait attributes by Microsoft (MS) Kinect image and depth sensors to track movements in three-dimensional space.

**Methods:**

The experimental part of the paper is devoted to the study of three sets of individuals: 18 patients with Parkinson’s disease, 18 healthy aged-matched individuals, and 15 students. The methodological part of the paper includes the use of digital signal-processing methods for rejecting gross data-acquisition errors, segmenting video frames, and extracting gait features. The proposed algorithm describes methods for estimating the leg length, normalised average stride length (SL), and gait velocity (GV) of the individuals in the given sets using MS Kinect data.

**Results:**

The main objective of this work involves the recognition of selected gait disorders in both the clinical and everyday settings. The results obtained include an evaluation of leg lengths, with a mean difference of 0.004 m in the complete set of 51 individuals studied, and of the gait features of patients with Parkinson’s disease (SL: 0.38 m, GV: 0.61 m/s) and an age-matched reference set (SL: 0.54 m, GV: 0.81 m/s). Combining both features allowed for the use of neural networks to classify and evaluate the selectivity, specificity, and accuracy. The achieved accuracy was 97.2 %, which suggests the potential use of MS Kinect image and depth sensors for these applications.

**Conclusions:**

Discussion points include the possibility of using the MS Kinect sensors as inexpensive replacements for complex multi-camera systems and treadmill walking in gait-feature detection for the recognition of selected gait disorders.

## Background

Systems that enable human–machine interactions [1, 2] and spatial modelling have a wide range of applications in modern engineering, robotics, and biomedical devices [3, 4].

**Fig. 1:**
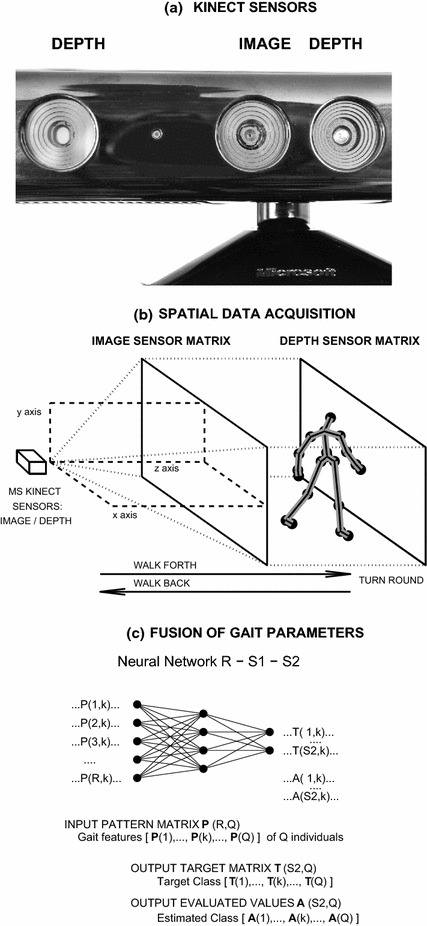
Data processing presenting **a** the location of the MS Kinect’s RGB camera and depth sensors, **b** the flowchart of spatial data acquisition in the given coordinate system, and **c** fusion of gait parameters to increase the classification accuracy

While complex synchronised video-camera systems represent precise but expensive technical solutions, it is possible to use much less expensive systems that employ depth sensors to acquire data with sufficient accuracy for many applications. Microsoft (MS) Kinect [4, 5] allows for the recording of such data sets via its image and depth sensors (illustrated in Fig. 1) and the subsequent transfer of these data to appropriate mathematical environments, such as MATLAB, for further processing. The acquired data sets can then be used to propose methods and algorithms for movement analyses [6], scene modelling [7], gesture and body recognition [8], rehabilitation [2], and posture reconstruction [9, 10]. These new devices, combined with motion sensors [11] and specific control units, are also often used for objective gait analysis.

This article is devoted to the use of the MS Kinect system for movement-data acquisition, the detection of gait features, and the analysis of gait disorders [12–15] via selected digital signal- and image-processing methods. The proposed graphical user interface was used to acquire clinical data from patients with Parkinson’s disease [16–18] and from healthy individuals who were used to form a reference dataset. Specific algorithms were then designed and used for motion tracking and gait-feature evaluation and for classification of the observed sets of individuals. The results were evaluated from both engineering and neurological perspectives.

The proposed methods show how modern sensors can be used to acquire data that enable human–machine interaction. The application discussed here is devoted to the use of MS Kinect as an alternative to treadmill walking in evaluating walking parameters and recognizing gait disorders [19, 20]. Signals and matrices acquired in this way can be further used in other applications, including rehabilitation engineering and robotic systems control.

Data classification and identification strategy constitute important parts of signal processing. There are many methods for data pre-processing, clustering and visualisation [21]. Different probabilistic methods including Bayesian methods, neural networks, and radial basis function units form the basis of many current software tools (e.g. Weka) [22]. The present paper applies some of these methods to the classification of gait features, along with the evaluation of the results and their cross-validation. Receiver operating characteristic (ROC) curves [23], along with sensitivity, specificity and confusion matrices, are used to analyse classification models.

MS Kinect sensors can record video frames and define time series of the movement of specific body parts [19] with sufficient accuracy in many cases. However, motion capture systems can be analysed from a wider point of view. Dynamic time warping methods can identify individuals on the basis of kinematic characteristics [24]. The distribution of spectral components of bodily movement allows for the design of smoothing filters [6], and spectral analysis can be used for the recognition of motion signals using accelerometers as well [25]. The study of Biovision hierarchical data and motion-capture based modelling provide additional tools for gait analysis [26].

## Methods

### Data acquisition

Information related to the bodily motions of the participants was recorded with MS Kinect sensors. The RGB camera recorded video image frames with a frequency of 30 fps. The depth sensor consists of an infrared projector and an infrared camera that uses the structured light principle [27, 28] to detect the distances between image pixels. Both the RGB camera and the depth sensors store information in 640 $\times$  480 element matrices. The accuracy of the system is fundamental for spatial data modelling [14, 15, 29] and, as expected, was in the range of $-$40 to 40 mm.

**Fig. 2:**
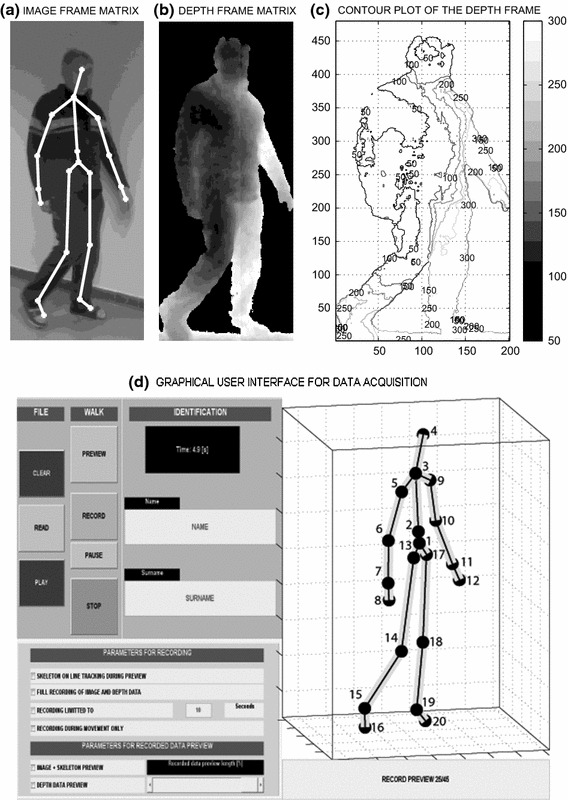
An example frame recorded by MS Kinect including **a** the image frame matrix combined with the skeleton estimate, **b** the depth frame matrix, **c** the contour plot of the depth frame matrix with distances from the selected plane, and **d** the proposed graphical user interface that was used to record MS Kinect data from the observed individuals in a clinical environment and to preview the recorded skeleton, video, and depth sensor data with numbering of joints

Figure 2 presents portions of selected frames that were recorded by the image and depth sensors. The selected image presented in Fig. 2a is combined with the skeleton projection and the estimated positions of the joints. Figure 2b, c illustrate information from the depth sensor. The contour plot in Fig. 2c presents the distances of the individual pixels from a selected (virtual) plane that was at a distance of 2200 mm from the MS Kinect.

The proposed graphical user interface (GUI) that was used to record MS Kinect data from the observed individuals, along with the processing of the obtained information in the MATLAB environment, are presented in Fig. 2d. The joint numbering presented in Table 1 is used. The GUI was designed to allow the simple recording of video frames in clinical environments via the following steps:

1. recording the name and surname of the patient;
2. initialising MS Kinect;
3. beginning the recording process by pressing the RECORD button, and initiating its interruption using the STOP button.

Further functions of the GUI included the selection of additional parameters for recording and data sets preview, including the options of previewing image and depth sensor data from the database. The skeleton-tracking algorithm, which processes these data, also provides information about the locations of joints, as specified in Fig. 2d. The joint numbering and the connection map are presented in Table 1.

**Table 1:** Skeleton positions and connection map of a standing individual that were used for data acquisition and video record processing

Skeleton positions			
Joint	No.	Joint	No.
Hip centre	1	Wrist right	11
Spine	2	Hand right	12
Shoulder centre	3	Hip left	13
Head	4	Knee left	14
Shoulder left	5	Ankle left	15
Elbow left	6	Foot left	16
Wrist left	7	Hip right	17
Hand left	8	Knee right	18
Shoulder right	9	Ankle right	19
Elbow right	10	Foot right	20

Connection map			
Part	Connection vectors		
Spine	[1 2], [2 3], [3 4]		
Left hand	[3 5], [5 6], [6 7], [7 8]		
Right hand	[3 9], [9 10], [10 11], [11 12]		
Left leg	[1 13], [13 14], [14 15], [15 16]		
Right leg	[1 17], [17 18], [18 19], [19 20]		

The notation “right/left” is related to the image and not to physical space (the subject’s body)

The experimental portion of this study was devoted to gait analyses of the three sets of individuals presented in Table 2, which included the following: (1) 18 patients (52–87 years of age, mean: 73.6, standard deviation: 9.2) with Parkinson’s disease (PD); (2) 18 healthy individuals (norm; 32–81 years of age, mean 55.0, standard deviation: 14.5) who formed the first reference set; and (3) 15 students (STUD), who formed the second reference set (23–25 years old, mean 23.7, standard deviation: 0.7). MS Kinect, which was used for data acquisition (as illustrated in Fig. 1), was installed approximately 60 cm above the floor. Each individual repeated 5 straight walks (segments) of approximately 4 m (5 steps) back and forth. Each video recording was acquired at a sampling rate of 30 fps. The video recordings contained both useful information about the direct walk and undesirable frames that were recorded while the individuals were turning.

### Skeleton tracking

The skeleton-tracking algorithm processed data matrices from the image and depth sensors and also provided coordinates that specified the spatial locations of all joints in the selected coordinate system [30], as illustrated in Fig. 1, by utilising the joint numbering and connection maps defined in Fig. 2d and Table 1.

The skeleton-tracking algorithm processed data in the four-dimensional field $\mathbf{T}(m,n,j,k)_{20,3,J,K}$, that was recorded for each frame $j=1,2,\ldots ,J$ in the selected segment $k=1,2,\ldots ,K$ of the straight walk with three coordinates *n* of each joint $m=1,2,\ldots ,20$ as specified in Fig. 2d and Table 1. Basic gait features were then evaluated as the Euclidian distances between selected positions using the associated differences *d*(*n*, *j*, *k*) of their coordinates by the relation

1 $$\begin{aligned} D\{d(n,j,k)\}= D(j,k)=\sqrt{\sum _{n=1}^3\;d(n,j,k)^2} \end{aligned}$$

for the selected frame *j* and segment *k*.

**Fig. 3:**
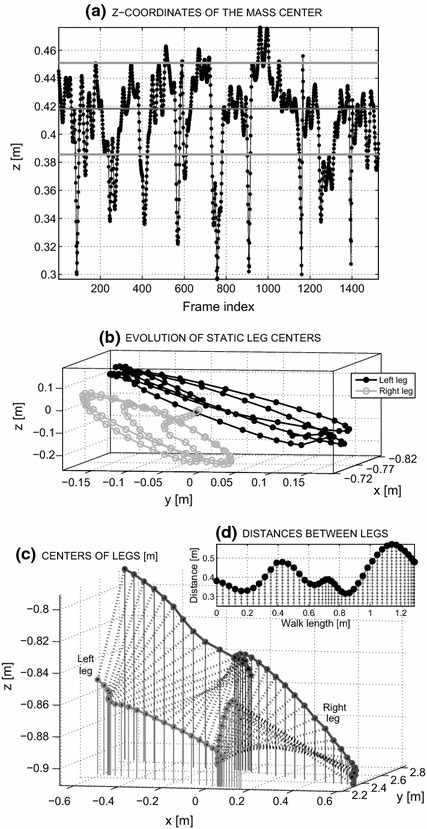
Visualisation of MS Kinect data presenting **a** the evolution of the z-coordinate of the COM in time with the median values and standard deviations used for the detection of gross errors and outliers rejection, **b** the relative spatial evolution of the left and right leg centres after the removal of the skeleton mass centre of each frame, **c** the temporal evolution of the right and left legs movement in three-dimensional space, and **d** the distances between the leg centres for a selected walk segment of a normal individual

The proposed algorithm for gait-features detection using MS Kinect can be summarised in the following steps:

1. *Rejection of frames with substantial errors* based on the time evolutions of the centres of mass of joints 1, 2, and 3 within the selected segment.
2. *Signal smoothing* with a selected filter that was applied to the time evolutions of all skeleton joints and the selection of data segments containing straight walking.
3. *Stride analysis* including detection of the leg lengths of all individuals from the skeleton data and stride-length estimation, based on the positions of the centres of legs (15, 16 and 19, 20) in each segment (Fig. 3b,c), with the Euclidian distances (Fig. 3d) of the leg’s centres followed by the detection of their maxima within a selected data segment.
4. *Gait-features estimation* of the following parameters: (1) the average step length of each individual in each segment of the straight walk normalised to the leg length of each individual and (2) the average speed of each individual.

*The rejection of frames with substantial errors* and outliers was related to the positions of the centres of mass (COM) within each frame, as evaluated based on three joints: shoulder centre, spine, and hip centre (i.e., 1, 2, 3). For each of these joints,

2 $$\begin{aligned} COM(n,j,k) = mean(\mathbf{T}([1~2~3],n,j,k) ) \end{aligned}$$

all coordinates, $n=1,2,$ and 3, in the selected frame *j* and segment *k* were evaluated. Fig. 3a illustrates the resulting evolution of the z-coordinates of the centres of mass (evaluated based on joints 1, 2 and 3) during a single experiment. The median value of the z-coordinate of each COM was used as the reference value, and frames with COM z-coordinates outside of the standard deviation limits shown in Fig. 3a were removed from the sequence of observations. Fig. 3b presents the relative spatial evolution of the left and right leg centres after the removal of the skeleton mass centre of each frame for a selected walk segment.

*Signal smoothing*  using the Savitzky–Golay filter [31, 32] was applied in the processing of individual skeleton joint positions. Each separate sequence

3 $$\begin{aligned} s_m(j)=\mathbf{T}(m,n,j,k) \end{aligned}$$

describing the evolution of the position of each joint *m* over frame index *j* (time) in the selected segment *k* was approximated by the Savitzky–Golay low-pass FIR filter by the sequence

4 $$\begin{aligned} \hat{s}_m(j) = \sum _{l=-L}^{L} a_l\,s_m(j-l) \end{aligned}$$

for all values of *j*. Filter coefficients $a_l$ were evaluated using the least-squares method [32] with the set of polynomials $p_j(l)$

5 $$\begin{aligned} p_j(l) = \sum _{r=0}^{R} c_r\,l^r \end{aligned}$$

of order *R* with their coefficients estimated using the least-squares method to minimise the error

6 $$\begin{aligned} E_R(j)= \sum _{l=-L}^{L}\left( p_j(l) -s_m(j+l)\right) ^2 \end{aligned}$$

for all values of *j*. A second-order Savitzky–Golay filter using 25 frames of overlap was used in this study to reduce errors in the estimations of joint positions.

**Fig. 4:**
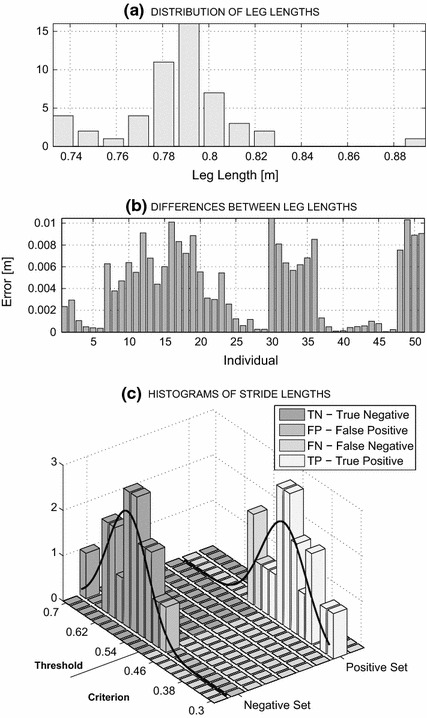
Results of the evaluation of leg lengths using MS Kinect during gait execution presenting **a** a histogram of the average leg lengths of separate individuals, **b** errors in the differences of the lengths of the left and right legs of individuals, and **c** the normalized stride length distributions for the individuals with Parkinson’s disease (positive set), the age-matched controls (negative set), and the distributions of true and false results across criterion values

*Stride analysis* represents the main processing step. To enable normalisation, the leg lengths of all individuals were evaluated first. By computing the differences between the left and right hip–knee and knee–ankle lengths, it was possible to estimate the length of each subjects left leg

7 $$\begin{aligned} D_{13-14}(j,k)= D\{T(13,n,j,k)-T(14,n,j,k)\} \end{aligned}$$

8 $$\begin{aligned} D_{14-15}(j,k)= D\{T(14,n,j,k)-T(15,n,j,k)\} \end{aligned}$$

9 $$\begin{aligned} LL(j,k)= D_{13-14}(j,k) + D_{14-15}(j,k) \end{aligned}$$

and right leg

10 $$\begin{aligned} D_{17-18}(j,k)= D\{T(17,n,j,k)-T(18,n,j,k)\} \end{aligned}$$

11 $$\begin{aligned} D_{18-19}(j,k)= D\{T(18,n,j,k)-T(19,n,j,k)\} \end{aligned}$$

12 $$\begin{aligned} RL(j,k)= D_{17-18}(j,k) + D_{18-19}(j,k) \end{aligned}$$

using the skeleton joint numbering detailed in Table 1. Each individual’s average

13 $$\begin{aligned} L(j,k)=(LL(j,k) + RL(j,k)) /2 \end{aligned}$$

was then evaluated for the selected frame *j* and segment *k*. The results of the evaluation of leg lengths over all frames and segments for all individuals are presented in Fig. 4a, b.

The Euclidian distance between feet was evaluated based on the average positions of the ankles and feet of the left leg (i.e., the average of the positions of joints 15 and 16) and the right leg (i.e., the average of the positions of joints 19 and 20)

14 $$\begin{aligned}  DIST(j,k) &= D\{\,mean (\mathbf{T}([15~16],n,j,k) ) \nonumber\\ &\quad - mean \, (\mathbf{T}([19~20],n,j,k) ) \} \end{aligned}$$

for each frame *j* and segment *k*. Relative maxima of these distances for a selected walk segment presented in Fig. 3c were used in evaluating the number of steps.

*The estimation of gait features* was based on all segments that contained walks in one direction and used the evaluated distances between the legs, normalised by the leg length of each individual. Projections of the movements in single coordinates were used for the following: (1) the detection of local extremes in that direction; (2) the identification of segments containing walks in one direction that occurred between the turns performed by the subjects, and (3) the rejection of the turn artefacts in each segment. The first and last local extremes were used to estimate gait velocity. The number of steps was defined as the number of extremes in this range.

The walking distances of the object in the first and last frames were evaluated as an Euclidian distance of the average joint positions over the whole skeleton. As the total number of steps was a result of the stride analysis, it was possible to identify the stride length in each segment by considering the ratio of the walking distance to the number of steps. The gait velocity was estimated as the ratio of the walking distance to the time difference between the first and last frames. The mean values for stride length and gait velocity over all segments were then considered as features of each subject.

### Gait features processing

Gait features were processed in order to classify the individuals in the selected data sets. The pattern matrix $\mathbf{P}_{R,Q}$ contained, in each column $q=1,2,\ldots ,Q$ the *R* features of each individual, which included (1) normalised stride length and (2) gait velocity. As the actual classification of each individual had previously been performed by a neurologist, it was possible to evaluate the selectivities, specificities, and accuracies of the positive set (i.e., individuals with Parkinson’s disease) and the negative set (i.e., the age-matched healthy individuals) for each of the selected features.

The feature histograms of the stride lengths (normalized to the average leg length) of the two populations presented in Fig. 4c were used for data classification. The estimation of the optimal stride length threshold for the identification of the subjects’ group memberships was determined in this stage as well. Neural networks were then used to classify features obtained. Further methods could include Bayesian classification [33].

#### Evaluation of classification results

Receiver operating characteristic (ROC) curves [23, 34, 35] provide an effective tool for analysing the features of normal (negative) and diseased (positive) individuals. The participants in this study formed two different true-negative and true-positive data sets. A selected classifier detects the following in the negative set (i.e., the controls):

- *TN* number of true-negative individuals, and
- *FP* number of false-positive individuals.

Similarly, the classifier identifies the following in the positive set (i.e., the patients with Parkinson’s disease):

- *TP* number of true-positive individuals, and
- *FN* number of false-negative individuals

Common performance metrics calculated from the confusion matrix include the following:

- *TP/FN rate* which is the probability of positive/negative classification within the positiveset:
  15 $$\begin{aligned} TPR=\frac{TP}{TP+FN}, \quad FNR=\frac{FN}{TP+FN} \end{aligned}$$
  defining sensitivity $$SE=TPR$$.
- *TN/FP rate* which is the probability of negative/positive classification within the negative set:
  16 $$\begin{aligned} TNR=\frac{TN}{FP+TN}, \quad FPR=\frac{FP}{FP+TN} \end{aligned}$$
  defining specificity $$SP=TNR$$.
- *Accuracy* which is the probability of obtaining the correct test result:
  17 $$\begin{aligned} ACCU=\frac{TP+TN}{TP+TN+FP+FN}. \end{aligned}$$

Cross-validation [36]  using  the leave-one-out scheme is often used to study the generalisability of proposed classification algorithms.

#### Neural networks use for classification

Combining both features allowed for the use of neural networks for classification and for evaluations of selectivity, specificity, and accuracy as well. The artificial neural network analysis [36] of the given set of *Q* individuals was based on the classification of *R* features that were recorded in the pattern matrix $\mathbf{P}_{R,Q}$.

The proposed classification algorithm used a two-layer neural network ($R-S1-S2$) with *R* input elements, sigmoidal transfer functions *F*1 and *F*2 in each of the layers and selected numbers of neurons in the first (*S*1) and second ($S2=2$) layers. The output values were evaluated for the weight matrices $\mathbf{W1}_{S1,R}$ and $\mathbf{W2}_{S2,S1}$ and threshold values $\mathbf{b1}_{S1,1}$ and $\mathbf{b2}_{S2,1}$ using the following relations:

18 $$\begin{aligned} \mathbf{A1}_{S1,Q}= F1( \mathbf{W1}_{S1,R}\; \mathbf{P}_{R,Q}, \; \mathbf{b1}_{S1,1} ), \end{aligned}$$

19 $$\begin{aligned} \mathbf{A2}_{S2,Q}= F2( \mathbf{W2}_{S2,S1}\; \mathbf{A1}_{S1,Q}, \; \mathbf{b2}_{S2,1} ). \end{aligned}$$

An associated matrix of target values $\mathbf{T}_{S2,Q}$ was formed by zeroes (for the reference individuals) and ones (for the positive individuals).

During the iterative learning process, the network weights were altered to minimise the distances between the evaluated network outputs and the target values in the least squares sense, using $60~\%$ of the feature vectors as the learning set, $20~\%$ as the validation set with which to test the end result of the learning process, and the final $20~\%$ to test the network’s behaviour. The results of the classification were evaluated via the confusion matrix, which shows the correctly classified values (i.e., the numbers of true-positive and true-negative individuals) on its diagonal. The off-diagonal values represent misclassifications and summarise the false-negative and false-positive individuals.

**Fig. 5:**
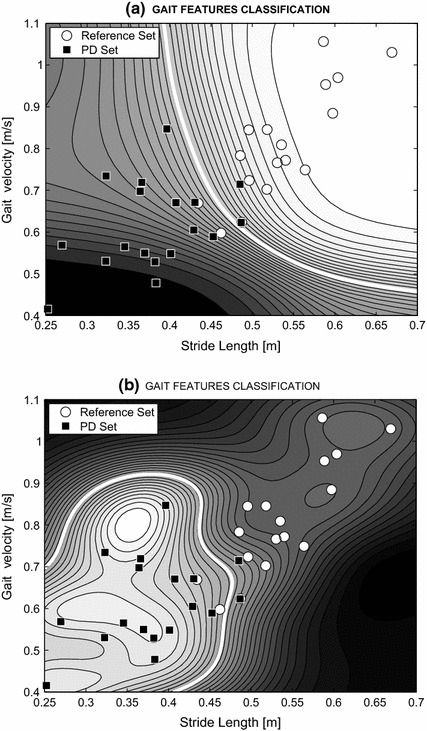
Gait features and their classification into two classes by **a** the two-layer sigmoidal neural networks 2-4-2 and **b** the RBF neural networks 36-18-1 with the spread of radial basis functions equal to 0.1

A two-layer sigmoidal neural network 2-4-2 was used for the classification of gait features (stride length and gait velocity) for 36 individuals, who were classified into two groups (controls and PD patients). Results are presented in Fig. 5a. The radial basis function (RBF) network 36-18-1 with 18 elements, which is presented in Fig. 5b, provides a much more sophisticated decision boundary allowing for the classification of much more complex clusters in general. Their design [37, 38] includes k-means clustering, definition of RBF activation functions, and training of the whole system.

## Results

**Fig. 6:**
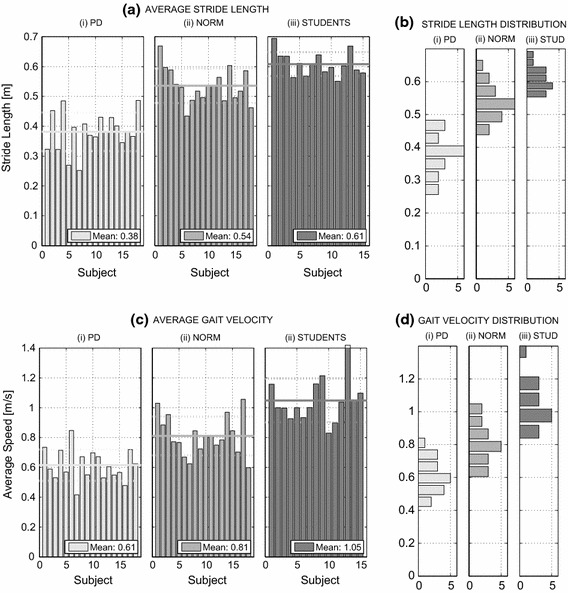
Selected gait features obtained from MS Kinect, including **a** the average stride lengths and corresponding standard deviations, **b** the histograms of the stride length distribution, **c** the average gait velocities and corresponding standard deviations, and **d** histograms of the distribution of the velocities for three sets of individuals (i.e. individuals with Parkinson’s disease—PD, age-matched controls—NORM, and the second reference sets of students—STUDENTS )

The patients examined had stage II or III Parkinson’s, according to the Hoehn and Yahr scale. The proposed methodology represents a pilot study for identifying the gait features having the most significant values. Most patients examined were well aided by the therapy, which made discrimination more difficult. Table 2 and Fig. 6 present descriptions of the data sets of the 18 individuals with Parkinson’s disease, along with the 18 controls and the 15 students. The numerical results obtained from data acquired by MS Kinect at a sampling rate of 30 fps using the proposed algorithm, after the reduction of observation errors, are also presented Table 2.

**Table 2:** Characteristics of three sets of individuals (PD: Parkinson’s disease, Norm: controls, STUD: students) and the results of their analysis including their average leg lengths (LL), stride lengths (SL) and gait velocities (GV) with their standard deviations (SD)

Group-size		Age (years)	LL (m)		SL (m)		GV (m/s)	
			Mean	SD	Mean	SD	Mean	SD
PD	18	52–87	0.785	0.034	0.38	0.07	0.61	0.12
Norm	18	32–81	0.783	0.027	0.54	0.06	0.81	0.15
STUD	15	23–25	0.792	0.009	0.61	0.04	1.05	0.15

The first goal of this study was *to estimate leg lengths* based on skeleton data from all 51 participants. Data plotted in Fig. 4a, b correspond to values averaged over all straight-walk segments. The mean difference between the right and left leg lengths of all individuals was 0.004 m (range 0–0.01 m; SD 0.003). The distribution of the average leg lengths of all 51 individuals is presented in Fig. 4a. The mean value was 0.786 m (SD = 0.026). The average leg length values for each group are presented in Table 2.

The second goal of this study was *to compare the PD set and the reference set*, as presented in Fig. 6. For comparison against the age-matched controls, two sets of features were used:

- The first feature was the average stride length obtained from the MS Kinect data, which was normalised to each individual’s average leg length. The resulting values for individuals with Parkinson’s disease (SL = 0.38 m, SD = 0.07) and the age-matched individuals (SL = 0.54 m, SD = 0.06) illustrate that, as expected, the average stride length of the PD group was shorter than that of the reference set.
- The second feature was the estimated gait velocity for all individuals. The results revealed a difference between the PD group (GV = 0.61 m/s, SD = 0.12) and the group of age-matched individuals (GV = 0.81 m/s, SD = 0.15).

Table 2 presents the results of the analyses based on the second reference set of healthy students, who had a lower average age of 23.7 years. The evaluated features illustrated in Fig. 6 (SL = 0.61 m, GV = 1.05 m/s) exhibit differences from both the PD group and the group of age-matched individuals. These results suggest that gait features are age dependent.

**Fig. 7:**
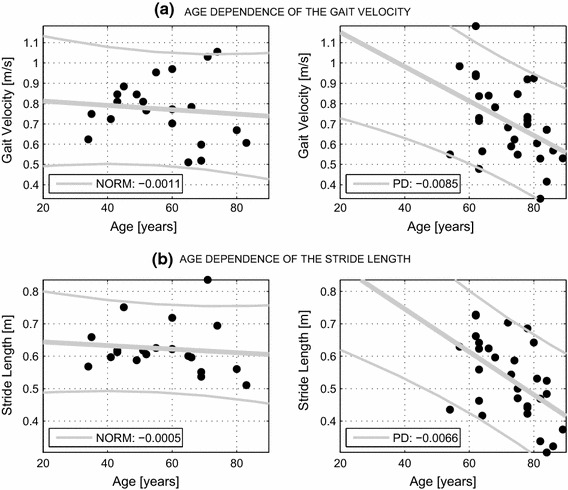
The age dependence of **a** the gait velocity and **b** the stride length with related regression coefficients

The study examining the age dependency [39] of selected gait features is presented in Fig. 7. The linear regression shows a decreasing trend in both features with age in the group of diseased subjects, but there is no age dependence for healthy individuals.

**Fig. 8:**
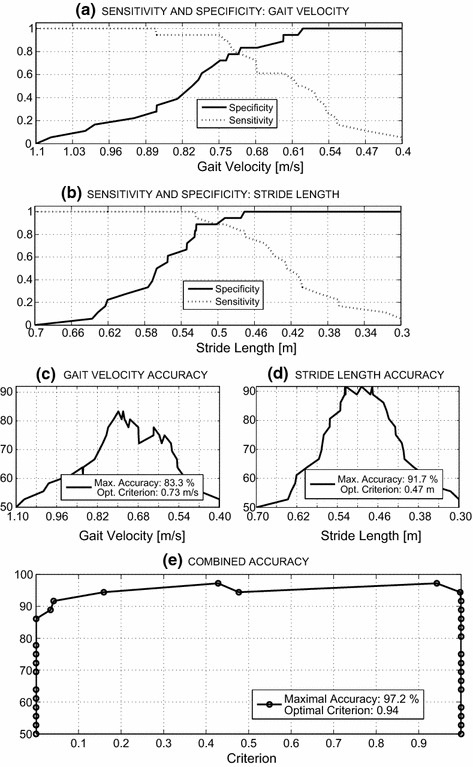
Sensitivity/specificity plots for the processing of **a** gait velocity, **b** stride lengths and classification accuracy presenting results for **c** the gait velocity features, **d** the stride length features, and **e** combined features processing using neural networks

*ROC analyses of the classifications* based on both single-gait features and the combination of gait features utilising sensitivity and specificity measures [35]—represented another goal of this study. Selected results are presented in Fig. 8. All curves presented were evaluated from Eqs. (15)–(17) for *TN*, *FP*, *TP*, and *FN* values dependent upon the criterion parameter according to Fig. 4c.

Using the true negative (TN)/false positive (FP) observations for the negative set (controls) and the true positive (TP)/false negative (FN) observations for the positive set (patients), it was possible to gauge the accuracy of each selected criterion (i.e., stride length or gait velocity) value. The results include the following findings:

- Analysis of the use of a single feature for classification revealed that an accuracy of 83.3 % could be achieved with an optimal gait-velocity threshold value of 0.73 m/s, as shown in Fig. 8c. The use of average stride length as a feature resulted in an accuracy of 91.7 % for the optimal stride-length value of 0.47 m, as presented in Fig. 8d. Cross-validation using the leave-one-out scheme resulted in a value of 0.25 for gait velocity and 0.139 for stride length.
- Combining the selected features of the pattern matrix for classification resulted in accuracies above 95 % for a wide range of criterion values, as shown in Fig. 8e. The use of the combination of features increased the range of reliable classifications compared to the use of single features.

The statistical results of the neural network classification are summarised in the following confusion matrix:

**Table:** 

	Target class		
	1	2	(%)
Output class			
1	*17 (47.2 %)*	0 (0.0 %)	*100.0*
			0.0
2	1 (2.8 %)	*18 (50.0 %)*	*94.7*
			5.3
	*94.4 %*	*100.0 %*	*97.2*
	5.6 %	0.0 %	2.8

Diagonal cells illustrate the numbers and percentages of correctly classified cases, while off-diagonal cells illustrate the misclassified cases. The cell at the bottom right shows that the classification was correct in 97.2 % of the cases and that a total of 2.8 % of cases were misclassified.

The confusion matrix specified above allows more detail analysis [23] of given sets, which implies $SE = 100$ % of true-positive and $SP = 94.4$ % of true-negative values using Eqs. (15)–(17).

**Table:** 

Confusion matrix		Performance matrix	
TN = 17	FN = 0	SP = 94.4 %	FNR = 0.0 %
FP = 1	TP = 18	FPR = 5.6 %	SE = 100.0 %

This result indicates that good recognition was achieved by using of the combination of gait features. Cross-validation using the leave-one-out scheme yielded a value of 0.083 (3 misclassified individuals out of 36) for the proposed neural network model.

Results achieved by the two-layer neural network 36-4-2 with sigmoidal transfer functions are compared with several further models in Table 3. It is possible to observe that radial basis functions allow for higher classification accuracy using a great number of neurons, but the generalization properties can be reduced owing to the decreasing value of the spread of radial basis functions. K-fold cross validation was performed for $Q=36$ individuals, $K=Q$ (the leave-one out cross-validation) and $K=Q/2$ with misclassified number of values given in brackets.

**Table 3:** Classification results of the set of *Q* = 36 individuals (18 individuals with the Parkinson’s disease and 18 controls) using two features (stride lengths and gait velocities) and different models with selected transfer functions (TF), spread (S) and misclassified number of values in brackets

Classification model	System parameters	Accuracy	K-fold cross validation	
			K = Q	K = Q/2
Perceptron	Hardlim	88.9	0.11 (4)	0.14 (5)
RBFN 36-7-1	Radial (S:1)	88.9	0.11 (4)	0.08 (3)
RBFN 36-18-1	Radial (S:0.1)	91.7	0.17 (6)	0.14 (5)
NN 36-4-2	TF: sigmoid	97.2	0.08 (3)	0.11 (4)

## Discussion

This paper outlines the possibility of using MS Kinect to measure gait features and to detect gait disorders caused by Parkinson’s disease. To normalise the stride lengths, the lengths of the participants’ legs were measured using MS Kinect. The processing of joint positions yielded in an average difference of 4 mm between the lengths of the left and right legs of the 51 individuals. This result indicates the high accuracy of the system, which corresponds to statistical observations suggesting that differences of up to 20 mm are considered medically normal and that this difference is greater than 5 mm in 60 % of the population.

The proposed method resulted in maximum classification accuracies of greater than 97.2 % for the given set of individuals with Parkinson’s disease and the age-matched controls. The confusion matrix indicates that a 97.2 % correct classification rate and a 2.8 % misclassification rate are sufficient for correct neurological classification.

Sigmoidal neural networks were used for the classification of gait features. It was found that radial basis function networks can achieve similar accuracy but their coefficients must be well chosen in order not to reduce the classification system’s ability to generalize. Cross-validation using the leave-one-out scheme resulted in error-rate values of 0.25 and 0.139 for gait velocity and stride length, respectively, while the combination of these features by the neural network model decreased the cross-validation to 0.083.

The second reference set of 15 students exhibited features differed from both the set of Parkinson’s patients and the group of age-matched controls. These findings suggest that the selected features were age-dependent, as has commonly been found in other areas of biomedicine.

## Conclusion

Human–machine interaction and computer intelligence belong to the rapidly developing interdisciplinary area that combines sensor technology, data fusion, computer vision, image processing, control engineering and robotics. Numerous papers have been devoted to the identification and detection of motion features [11, 40] with applications in biomedical signal processing and the diagnosis of gait disorders [17].

Motion analysis and Parkinson’s disease recognition can be performed by specialised and expensive camera systems with specific sensors. These systems are commonly used for the detection of movement with high accuracy. This paper has presented a new approach to analysing gait disorders that utilises the inexpensive MS Kinect device. MS Kinect has a depth-sensor accuracy of 4–40 mm, which is sufficient for many applications. The results obtained suggest the possibility that MS Kinect can be used for the detection of gait disorders and for the recognition of Parkinson’s disease. The maximum accuracy observed in the present study was 97.2 %. It is assumed that classification of gait features will be used to observe the effects of medication and rehabilitation.

Further work will be devoted to the study of more extensive data sets and to the evaluation of a higher number of parameters, with the goal of more accurately classifying motion features across a wide range of criterion values. We assume that the synthesis of data from an increased number of biosensors will produce pattern matrices that can be used to give more accurate classification across a wide range of criterion values and provide tools for remote diagnostics and wireless data processing.

## Consent

All patients signed the informed consent to participate in the project with all the procedures approved by the Local Ethics Committee.
